# Itch in Chronic Wounds: Pathophysiology, Impact, and Management

**DOI:** 10.3390/medicines6040112

**Published:** 2019-11-15

**Authors:** Michela Iannone, Agata Janowska, Valentina Dini, Giulia Tonini, Teresa Oranges, Marco Romanelli

**Affiliations:** 1 Department of Dermatology, University of Pisa, 56126 Pisa, Italy; agatina82@gmail.com (A.J.); valentinadini74@gmail.com (V.D.); giuliatonini19@gmail.com (G.T.); teresa.oranges@gmail.com (T.O.); romanellimarco60@gmail.com (M.R.); 2 Department of Health Sciences, Anna Meyer Children’s University Hospital, University of Florence, 50139 Florence, Italy

**Keywords:** chronic pruritus, itch, pruritus, wounds, itch in wounds, itch management

## Abstract

**Background:** The aims of this review are to analyze the current literature regarding the characteristics and pathophysiological mechanisms of itch in chronic wounds, to assess the impact on quality of life and delayed-healing, to focus on the best strategies of prevention and treatment, to highlight the importance of on-going research in order to fully understand the pathophysiology, and to improve the management of target therapies. **Methods:** A systematic literature review was performed using MEDLINE, PubMed, Embase, Scopus, ScienceDirect, and the Cochrane Library. We included a total of 11 articles written in English with relevant information on the pathophysiology of itch in chronic wounds and on management strategies. **Results:** Itch in chronic wounds was found to be correlated with xerosis, larger wound areas, necrotic tissue and amount of exudate, peripheral tissue edema, sclerosis, granulation tissue, contact dermatitis, and bacterial burden, as well as with lower quality of life. **Conclusions:** Although there are several aspecific pharmacological and non-pharmacological approaches, there appears to be no validated prevention or management strategy for itch in chronic wounds. Further studies are needed to clarify the association and pathophysiology of itch in chronic wounds, to evaluate the safety and efficacy of topical treatments on perilesional skin to reduce itch, to characterize multidimensional sensations of itch in chronic wounds, to identify specific cytokine and chemokine expressions that are correlated to a tailored-based approach, and to develop practical guidelines.

## 1. Introduction

Itch is a chief symptom in many dermatological diseases, which significantly impacts patients’ quality of life (QoL) [1]. Few studies, however, have analyzed the clinical itch characteristics and pathophysiological mechanisms of itch in chronic wounds [2,3,4,5,6,7,8,9,10,11,12]. Thus, the aim of this review is to analyze the current literature on the characteristics and pathophysiological mechanisms of itch in chronic wounds, to assess the impact on QoL and delayed wound healing, and to focus on prevention and treatment strategies for pruritus associated with chronic wounds. 

## 2. Methods

### Literature Search

A systematic literature search was performed to identify major findings on itch in chronic wounds in adults. We used the following databases: MEDLINE, PubMed, Embase, Scopus, ScienceDirect, and the Cochrane Library. The search included all studies published between January 2000 and June 2019. Keywords used were: itch in wounds, itch in leg ulcers, itch, chronic venous disease, wound pruritus, chronic wound itch, and itch management. We included only articles in English, with relevant information on the pathophysiology of wound-related itch and on management strategies. We excluded case reports, pediatric articles, and articles on acute wounds such as post-burn wounds.

We included a total of 11 articles.

**Figure 1:**
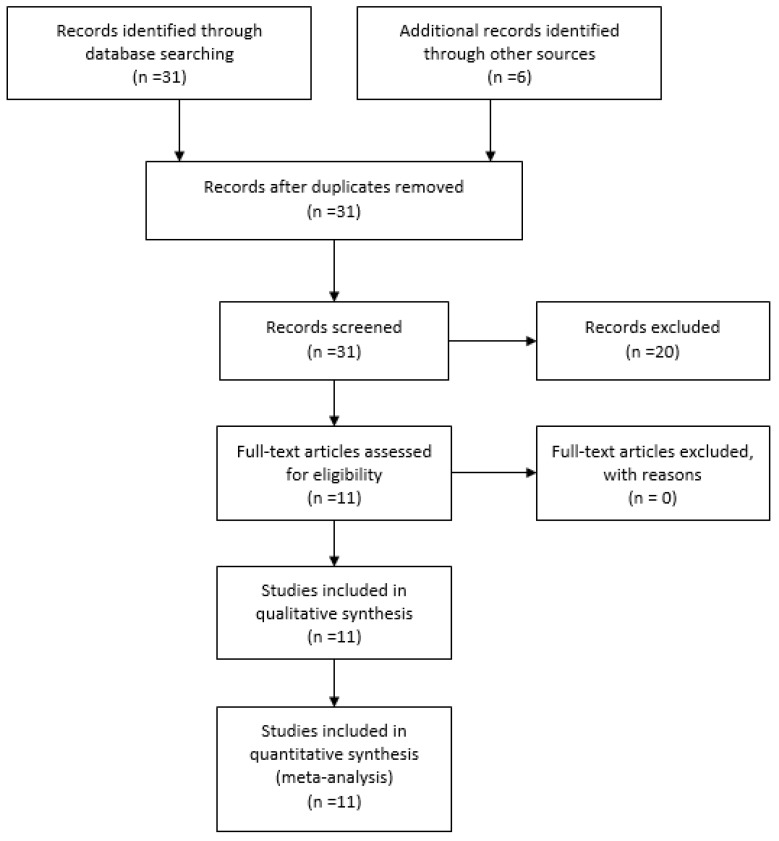
PRISMA flow diagram showing the literature search on itch in wounds.

The PRISMA 2019 flow diagram shown in Figure 1 explains the search methodology used in the study.

## 3. Results

### 3.1. Characteristics and Pathophysiological Mechanisms of Itch

We selected nine articles focused on the characteristics and pathophysiological mechanisms of itch. Table 1 summarizes the main key data—authors, year of publication, country, type of article, purpose of the study, and findings [4,5,6,7,8,9,10,11,12].

### 3.2. Impact on QoL

We selected four articles regarding the impact on QoL. The key data are summarized in Table 1 [2,3,4,7].

### 3.3. Prevention of Itch in Chronic Wounds 

We found no articles on how to prevent itch in chronic wounds, so we decided to correlate data on the pathophysiological mechanisms of itch with current wound care management strategies.

## 4. Discussion

Cutaneous chronic wounds are classified as vascular (arterial, venous, mixed arterial-venous), diabetic foot ulcers, pressure ulcers, and atypical wounds (such as inflammatory, neoplastic, vasculitis, and exogenous). Wound itch is a frequent problem in clinical practice, but is poorly described in the literature. There are currently no exact data on the incidence and/or prevalence of itch in cutaneous wounds. The only data available report the characteristics of wounds and their relationship with itch. Our results from the systematic review show a linear correlation between wound area and itch through the release of itch triggers such as histamine and growth factors on the wound bed [6]. 

Remaining on wound characteristics analysis, the amount of necrotic wound bed tissue is another important finding; dead tissue blocks healing and leads to scratching, with further damage and enlargement of wounds [6].

A high amount of exudate is another wound characteristic that causes maceration and is an itch trigger factor. The collection of fluids in tissue can also causes mechanical stress that may exacerbate itch and promote mast cell invasion into nerve fibers, which can trigger or aggravate itch [6]. 

The induration in the periwound area, i.e., sclerosis, is another potential cause of wound itch; tissue damage activates inflammatory processes with mast cell degranulation promoting the release of pruritogen mediators [7]. 

The final findings of our review are about the granulation tissue. This tissue occurs in the proliferative phase of the wound healing process and contains fibroblasts and different types of inflammatory cells and may also release neoangiogenesis factors, connective proteins, nerve growth factors, and pruritogen mediators, which partially explain the phrase “it’s itching, it must be healing”, commonly used by healthcare providers [6]. However in some conditions, such as in infected wounds, granulation tissue can be hypertrophic and friable, and can cause excessive itch. Infected wounds may also itch because bacterial biofilm can interact through proadrenomedullin N-terminal 20 peptide (PAMP) with Toll-like 2 receptors (TLR-2, and activate protein cascades with the release of itch mediators [13].

Regarding management, the tissue debridement, inflammation/infection, moisture imbalance, epithelial edge advancement (TIME) principles of wound bed preparation are particularly effective in the management of these pathophysiologic factors in order to reduce the itch sensation [14].

By correlating the level of itch with wound management, our literature review has shown that, in selected patients, moderate compression bandaging can be used to manage itch by increasing the venous tone and normalizing circulation by removing edema [15].

Another important itch management strategy is the proper care of perilesional skin by two steps: proper selection of the wound dressings in line with the level of exudate and the size of the wound and the utilization of barrier products (principally zinc oxide paste, silicone-based ointments, polymer barrier preparations) and moisturizers [16].

If causative treatment fails, a stepwise therapeutic approach based on the European S2k Guideline on Chronic Pruritus is recommended. Step 1 consists of moisturizers and emollients containing urea (5%–10%), glycerol (20%), camphor (2%), menthol (1%), zinc (10%), pramoxine (1%), and polidocanol, and in systemic therapies with anti-h1 non-sedating antihistamines. Step 2 consists of topical anti-inflammatories (steroids and calcineurin inhibitors), gabapentinoids, and mu-opioid receptor antagonists. Step 3 consists of adding selected antidepressants (paroxetine, mirtazapine, doxepin, amitriptyline) or neurokinin receptor 1 antagonists [17]. 

## 5. Conclusions

Itch in wounds is a very frequent symptom and should never be underestimated. A better characterization of itch in chronic wounds and the identification of best strategies of prevention and treatment would improve the daily functions, the psychological state, and the social interactions of patients affected by chronic wounds. 

The pathophysiology is particularly complex and multifactorial, and it is not fully understood. Numerous factors influence itch such as wound area, necrotic tissue amount, exudate amount, peripheral tissue edema, sclerosis, granulation tissue, bacterial biofilm, chronic venous insufficiency (CVI), perilesional skin characteristics, neuropathic changes, and dressing sensitization, as well as by psychological and emotional components. An itch-scratch cycle can lead to secondary infections, changes in pigmentation, thickening of the skin, and delayed healing.

The subjective and multidimensional nature of itch makes it a real challenge for clinicians. Various assessment tools have been used to evaluate itch. A critical point of further research is a consensus on the development of structured questionnaires to evaluate and measure the sensory and affective dimensions of itch in chronic wounds.

Currently, there are no standards for preventing and managing itch in chronic wounds. The TIME principles of wound bed preparation, the topical management of perilesional skin, and a stepwise therapeutic approach based on European S2k Guideline on chronic itch (if causative treatment has failed) seem to be the best management strategies to date.

Our study presents some methodological limitations. First, the literature data on the physiopathology and management of itch in chronic wounds was particularly poor. Second, itch has very complex underlying mechanisms of a subjective and multidimensional nature, which made our investigation complicated. Third, our literature review was limited to data available on online databases. 

Further studies are needed to clarify the association and pathophysiology of itch in chronic wounds, and to evaluate the safety and efficacy of topical treatments on perilesional skin and of moderate compression to reduce itch. Further research on correlations among severity of itch and cytokines, chemokines, and inflammatory marker levels in exudates, perilesional, and lesional skin in different healing phases would help in developing targeted therapies for itch in chronic wounds. 

Such studies should adopt a tailored-based approach and draw up practical guidelines.

The take-home messages of this review are summarized in Table 2.

## Figures and Tables

**Table 1:** Key data from literature review.

Authors	Year	Country	Type of Article	Purpose of Study	Findings
**Hareendran A. et al. [2]**	2005	United Kingdom	Qualitative and quantitative methods were used to explore health related quality of life (HRQoL) issues in 38 patients	Identify HRQoL issues in patients with venous leg ulcers	Itching was reported in 69.4% of patients but no influence on sleep and functional limitations were found
**Hareendran A. et al. [3]**	2007	United Kingdom	In-depth interviews with focus group of 36 patients	To develop and validate a disease-specific quality of life (QoL) measure, based on the conceptual model of the Skin Disease impact on quality of life Index (SKINDEX-29) for patients with chronic venous leg ulcers	Itch was ranked 4th among ten symptoms causing distress in chronic venous ulcers
**Paul J.C. et al. [4]**	2011	Michigan (USA)	Cross sectional study on 161 patients	Investigate itch related to chronic venous disease, pain, and QoL	Positive correlation between intensity of itch and severity of venous disease with lower QoL
**Paul J. [5]**	2013	Michigan (USA)	Cross sectional study on 199 patients with chronic wounds of different etiologies	Comparing pain and itch in chronic wounds	Wound-related itch was significantly associated with age, xerosis, employment status, and with venous wounds. Itch was rated higher on the perilesional skin, while pain was rated higher on the wound bed.
**Paul J. [6]**	2013	Michigan (USA)	Observational study on 200 patients with chronic wounds of different etiologies	Explore characteristics of wound-related itch	Itch characterizes more severe wounds with larger size, more tissue edema, and granulation issue and was also associated with moderate exudate amount or necrotic tissue
**Upton D. et al. [7]**	2013	United Kingdom	Literature review	Overview of the physiological mechanisms of itch and comorbidities in acute and chronic wounds	The itch causes a range of physical and psychological problems, reducing QoL and delaying healing. There are no specific guidelines on itch management in chronic wounds and further studies are needed.
**Upton D. et al. [8]**	2013	Australia	Literature review	Overview on psychological itch treatment in wounds	Unconventional treatments such as habit reversal training, relaxation, massage, and itch coping programs showed a potential role in reducing itch in association with standard treatments, but current literature evidence is limited.
**D’Erme A.M. et al. [9]**	2016	Italy	Literature review	Overview on contact allergy and polysensitization in patients with chronic wounds	Advanced dressings can cause allergic contact dermatitis. The most frequent was hydrogel, followed by hydrocolloid and by silver wound dressings. Primary prevention is required, avoiding sensitizers and irritant products, along with secondary prevention using patch tests in all patients with non-healing wounds.
**Paul J. [10]**	2018	Michigan (USA)	Structured interviews of 199 patients with chronic wounds	Identify descriptors for wound-related itch	15 descriptors identified (annoying, bothersome, just want itching to go away, unpleasant, stubborn, disturbing sleep, insistent, disgusting, severe, awful, prickly, warm, worrisome, unbearable, uncontrollable)
**Parnell L.K.S. et al. [11]**	2018	Texas (USA)	Literature review	Overview on itch research focusing on itch in wound care	Importance of multidimensional questionnaires to characterize itch. The authors described sensory, affective dimensions of itch, the itch trigger, and itch receptors and pathways. They highlighted both conventional and unconventional pharmacological therapies.
**Lerner E. [12]**	2018	South Carolina (USA)	Literature review	Overview of current understanding on the physiology of itch in wounds	Proposal for unconventional therapeutic approaches based on physiology

**Table 2:** Take-home messages.

Take-Home Messages
✓ Itch in wounds is a very frequent symptom and should never be underestimated. The underlying mechanisms are very complex, including those of a subjective and multidimensional nature, which make investigations a real challenge for clinicians.
✓ The application of the tissue debridement, inflammation/infection, moisture imbalance, epithelial edge advancement (TIME) principles of wound bed preparation, the topical management of perilesional skin, and a stepwise therapeutic approach based on European S2k Guideline on chronic itch (if causative treatment fails) seem to be the best management strategies to date.
✓ Further studies are needed to better characterize and develop targeted therapies for itch in chronic wounds, adopting a tailored-based approach and drawing up practical guidelines.
